# Molecular Diagnosis of COVID-19; Biosafety and Pre-analytical Recommendations

**DOI:** 10.30699/IJP.2023.1988405.3061

**Published:** 2023-07-16

**Authors:** Mohammad Vasei, Elham Jafari, Vahid Falah Azad, Moeinadin Safavi, Maryam Sotoudeh

**Affiliations:** 1 *Molecular Pathology and Cytogenetics Division, Pathology Department, Children's Medical Center, Tehran University of Medical Sciences, Tehran, Iran*; 2 *Cell-Based* *Therapies Research Center, Digestive Disease Research Institute, Shari'ati Hospital, Tehran University of Medical Science, Tehran, Iran*; 3 *Pathology and Stem Cells Research Center, Kerman University of Medical Sciences, Kerman, Iran*

**Keywords:** bio-safety, pre-analytical, sampling, transportation, diagnosis, COVID-19

## Abstract

From the beginning of the COVID-19 epidemic, clinical laboratories around the world have been involved with tests for detection of SARS-CoV-2. At present, RT-PCR (real-time reverse transcription polymerase chain reaction assay) is seen as the gold standard for identifying the virus. Many factors are involved in achieving the highest accuracy in this test, including parameters related to the pre-analysis stage. Having instructions on the type of sample, how to take the sample, and its storage and transportation help control the interfering factors at this stage. Studies have shown that pre-analytical factors might be the cause of the high SARS-CoV-2 test false-negative rates. Also, the safety of personnel in molecular laboratories is of utmost importance, and it requires strict guidelines to ensure the safety of exposed individuals and prevent the virus from spreading. Since the onset of the outbreak, various instructions and guidelines have been developed in this field by the institutions and the Ministry of Health of each country; these guidelines are seriously in need of integration and operation. In this study, we try to collect all the information and research done from the beginning of this pandemic in December 2019 - August 2022 concerning biosafety and protective measures, sample types, sampling methods, container, and storage solutions, sampling equipment, and sample storage and transportation for molecular testing of SARS-CoV-2.

## Introduction

The onset of the coronavirus infection was a global threat, and the outcome was a terrible pandemic. The outbreak of what was then named COVID-19 was caused by SARS-CoV2 (severe acute respiratory syndrome coronavirus 2) (1). The disease has multiple symptoms including shortness of breath, diarrhea, headache, fever, cough, myalgia, fatigue, sore throat, anosmia, chest pain, ageusia, hemoptysis, rhinorrhea, sputum production, skin rash, nausea, vomiting, impaired consciousness, and seizure (2).

Despite the existence of various methods in supportive care interventions and widespread vaccines to control and reduce the number of COVID-19 cases, the emergence of new strains of the virus has challenged the effectiveness of these measures. Hence, preventing the spread of the disease through diagnosis and isolation of the affected population greatly relies on the rapid and accurate detection of the virus (3, 4). Laboratories employ molecular diagnostic techniques to quickly and accurately detect the coronavirus to isolate it (5). Using sing NAAT (nucleic acid amplification test) for viral RNA detection is recommended to diagnose SARS-CoV-2. This is mostly done as reverse transcription (RT)-PCR tests performed on samples from the lower and upper respiratory tract (6). It remains to be determined if throat washing, saliva, stool, urine, or blood may also be acceptable replacements for these samples (7).

There is now incontrovertible evidence that in virology tests, as in other types of testing carried out for both diagnostic and research purposes, the pre-analytical phase is the main stage where errors occur (8). Pre-analytical errors comprise 60% of the errors of most diagnostic processes, and the analysis itself and the post-analytical phase are relatively error-free in comparison (9).

Payne* et al.* hypothesized that pre-analytical factors might be the cause of the high SARS-CoV-2 test false-negative rate (8, 9). The pre-analytical aspects of the test include failing to identify the patient and/or sample, inadequate quality or volume of collected specimen, inappropriate sample storage and transportation conditions, problematic materials released as a result of use of inappropriate additives or due to the whole blood-freezing, injury exposure, unreliable cold chain, too late or too early specimen collection (*e.g*., in the case of detection failure in late infections with atypical manifestations), manual errors, such as the use of wrong swabs leading to the inadequate absorption of diagnostic material, sample contamination, use of inadequate vials, and unreliable results due to the patients' having received antiretroviral therapy (4, 6, 9). So, this review aimed to highlight molecular diagnosis, biosafety, and pre-analytical recommendations for COVID-19 disease.

**Test Selection and Request Form**

Electronic test requisition is preferable to manual paper requests wherever possible, *e.g.* if the hospital uses a hospital information system (HIS) (10). When faced with a case suspected of COVID-19, it is necessary to record their complete name, date of birth, sex, address, mobile phone number, ID number, date of symptom onset, specimen type, date of sample collection, the test required, healthcare worker name, and similar important information on the case investigation form (CIF) and the associated request documentation, preferably immediately before sampling (11). 

**Specimen Type and Collection Methods**

Laboratory tests for diagnosis COVID-19 include rapid molecular tests, antibody tests, antigen tests, and self-collected respiratory sampling. 

The RT-PCR test is a common method for SARS-CoV-2 diagnosis (12). This test is used to confirm rapid diagnosis in symptomatic patients or to screen asymptomatic individuals (13). However, this diagnostic method produces many incorrect results such as false negatives (12). For cases that aim to screen asymptomatic individuals, pooled tests using rRT-PCR can be used. The rapid molecular test is used as rapid screening in emergencies in cases in which seven days have passed since the symptomatic disease, in cases that need confirmation of past infection or sero-surveillance, or in cases of multisystem inflammation syndrome. Other used tests include antigen and antibody tests (13). SARS-CoV-2 can also be detected through nucleic acid amplification test (NAAT). Many NAAT methods exist, including isothermal amplification tests (IATs) and RT-PCR (RT-qPCR, RT-ddPCR, and tests based on rapid RT-PCR) (4).

Antibody and antigen tests for the detection of SARS-CoV-2 include rapid antigen tests (Ag-RDTs) that detect SARS-CoV-2 based on immune response (proteins and antibodies) to it (4). 

There were two types of sampling: respiratory tract specimens and non-respiratory specimens. The respiratory tract specimens included nasopharyngeal swabs (NPSs), nasal swabs, oropharyngeal swabs, saliva, bronchial washings, bronchoalveolar lavage (BAL), endotracheal aspiration, sputum, and upper and lower respiratory tract samples, and the non-respiratory samples included blood, stool, and other fluids of the body (cerebrospinal fluid and urine) (14, 15). However, whether blood, saliva, conjunctival swabs, stools, cerebrospinal fluid (CSF), rectal swabs, and semen may also contain the virus remains undetermined (16). COVID-19 diagnosis and screening can be done by molecular detection in saliva or nasal swabs, but there is little merit in using other non-respiratory samples to detect COVID-19 (14). This is why we have little information on the frequency and duration of COVID-19 virus shedding in urine and stool (17). 

Research shows that COVID-19 management greatly relies on appropriate timing. In the early period (0 to 5 days), upper respiratory tract samples are more accurate in diagnosis the disease, but as the disease progresses, lower respiratory tract sampling may be associated with a higher diagnostic accuracy (18). 

Considering its greater sensitivity, facility of quantitative analysis, and better specificity in comparison to conventional RT-PCR, rqRT-PCR (real-time quantitative reverse transcription-PCR) has recently risen in popularity in the detection of coronavirus. Researchers have tried to improve real-time RT-PCR using a wide range of methods (19). The WHO interim guidance recommends the collection and testing of both upper and lower respiratory samples, such as sputum and bronchoalveolar lavage fluid (BALF), as they are the most sensitive detection methods of SARS-CoV, MERS-CoV, and SARS-CoV-2 (20). In case of high suspicion despite negative PCR results in the initial upper respiratory sample, it is suggested by the IDSA panel that instead of re-sampling the upper respiratory tract, a tracheal aspirate, bronchoalveolar lavage fluid, or sputum be collected. The certainty of evidence supporting this conditional recommendation is very low (21). 

Sutjipto* et al.* studied 105 patients, 73 of whom had active SARS-CoV-2 infections. They found that in the primary stages of the infection nasopharyngeal specimens present the highest sensitivity for the diagnosis of COVID-19, and the second rank in sensitivity belonged to throat samples (22). When throat specimens are used alongside nasopharyngeal or mid-turbinate specimens, there is an improvement in clinical sensitivity. In case the lungs have pneumonia or if the samples are collected one week after the illness, the specimens from the upper respiratory tract yield poor results in terms of diagnosis (22). However, in Yang* et al.*'s study, throat swabs were not recommended for virus detection. In severe and mild cases, the sputum samples, nasal swabs, and throat swabs showed 88.9% and 82.2%, 73.3%, and 72.1%, and 60.0% and 61.3% positive results, respectively (23).

In addition, a study revealed that the polymerase amplification (RT-RPA) method has been introduced as an ultrahigh-sensitive method in disease diagnosis (24).

A variety of swabs and transport media types and also unilateral versus bilateral nares self-sampling have been discussed in studies, but the panel rated the certainty of the evidence as low because in the same population, there weren’t any direct comparisons between samples of different types (25).

At present, upper respiratory specimens, especially those collected from the nasopharyngeal area, provide the best swab-based SARS-CoV-2 test results, especially in those with mild or no symptoms (26). Nasal aspirate or nasopharyngeal wash/aspirate specimens, spun polyester or flocked swab nasal (anterior nares) specimens, flocked tapered swab nasal mid-turbinate specimens, or oropharyngeal specimens are other alternatives when nasopharyngeal swabs cannot be used. Lower respiratory tract specimens (in BALF) are also viable options for individuals having invasive procedures (23). 

Regarding biosafety, nasopharyngeal sampling is preferred because the risk for healthcare workers is increased due to the production of aerosol droplets during the collection of sputum and BAL during bronchoscopy (27); Studies have also shown that oral fluid and saliva have sensitivities similar to nasopharyngeal swabs (NPS), and they have recently been suggested as potential methods that enable patients to collect the sample themselves (28, 29).

Printed pamphlets and recorded video instructions may be provided to facilitate self-collection, and patients can be informed. Healthcare workers have been at hand to aid self-collection research in the majority of cases.

Yet, there is very little data on self-collection in individuals without symptoms (21). However, there is a need for more studies comparing non-invasive self-collected samples, such as saliva and throat, mid-turbinate, and nasal swabs with nasopharyngeal swabs collected by healthcare providers. Studies comparing specimens collected at different times starting from the onset of symptoms, two-sided specimen collection *versus* on-sided collection, the amount of virus recovered through various methods, and evaluation of swabs collected from children with COVID-19 by parents would be extremely useful (21). eNAT (Copan Diagnostics Murrieta, CA) sterilizing buffer and saliva can be used safely and accurately for sensitive detection of the coronavirus by point-of-care GeneXpert instruments (30). New methods of self-collection and self-testing can provide more convenient, efficient, safe, and potentially cost-effective healthcare delivery for disease diagnosis and treatment (31). Accurate COVID-19 testing depends on the appropriate sample type, host factors, the elapsed since the beginning of symptoms, and the sample processing and collection methods (14, 16).

**Turnaround time (TAT) and Repeat Testing**

It is preferable to announce the results 24 hours after collection in the hospital. If necessary, the test should be repeated 1 to 2 days after the first test. If symptoms of lower respiratory tract infection are observed, it is advisable to collect samples from the lower respiratory tract in case the initial NAAT results return negative (32). Studies have shown that approaches with a high number of tests and short TAT are most effective in diagnostic tests. Modeling in these studies has shown that reducing testing delays has the highest impact on reducing subsequent transfers and optimizing testing coverage (33, 34). Therefore, TATs that may last several days decrease the effectiveness of isolation quarantine aiming to reduce disease transmission.

**Sampling from Asymptomatic Individuals**

Testing of asymptomatic individuals can play an important role in slowing transmission and controlling COVID-19, as nearly half of positive SARS-CoV-2 test results are in those who did not report symptoms at the time of testing (35). The recommendations of the IDSA panel state that before initiation of immunosuppressive procedures such as cytotoxic chemotherapy, solid organ or stem cell transplantation, and long-acting biologic therapy, cellular immunotherapy, or high-dose corticosteroids in individuals who are asymptomatic, SARS-CoV-2 RNA testing be done. Nevertheless, SARS-CoV-2 RNA testing is advisable within 48–72 hours of the planned treatment or procedure (as close to the procedure as possible) when there is limited access to PPEs (personal protective equipment), whether exposure to COVID-19 has been determined or not, for patients approaching time-sensitive aerosol-generating procedures, such as bronchoscopy, and for patients awaiting major time-sensitive surgeries (21). 

**Upper Respiratory Tract Specimens (Nasopharyngeal and Oropharyngeal Swab, Saliva, and** **Throat Washing)**

Coughing and sneezing induced by the collection of nasopharyngeal or throat swabs generate aerosols and have a high potential hazard for healthcare workers. The collection of throat swabs requires the healthcare worker to examine the tonsils and posterior pharynx of the patient directly. Furthermore, the possibility of bleeding and the discomfort that nasopharyngeal sample collection may cause makes it somewhat invasive (36). 

**Nasal swab: **After inserting the swab 3–4 cm parallel to the palate, we increase secretion absorption by rotating it for 5 to 10 seconds before performing the same procedure with the same swab for the other nostril and placing it in a capped tube and into a collection bag (37, 38). A study also showed that combined oropharyngeal and nasal swab studies can have more accurate results (39). Lee* et al.* in their study demonstrated that because of more sensitivity of nasal cavity swabs, this method can be an alternative method for the rapid diagnosis of SARS-CoV-2 (40). 

**Nasopharyngeal (NP) samples:** A polyester-tipped swab is used on a thin wire. The patient’s head is tilted back 70 degrees. The swab is held like a pen and passed along the floor of the nose through the nostril corresponding to the patient’s dominant hand (8–10 cm deep in adults) until it reaches the nasopharynx posterior wall and can go no further. Then the swab is gently rubbed and rolled several times before it is withdrawn (39, 41). If the tip has completely absorbed the fluid from the first sampling, collecting samples from both sides is not necessary. In case of blockage or septum deviation, the sample is obtained from the nasal fossa using the same swab (42). Patriquin* et al.* showed that nasal swabs have many benefits for detecting SARS-CoV-2, but they are less sensitive compared to a normal nasopharyngeal swab (41).

**Nasopharyngeal aspirates**: Catheter tubing is connected to a syringe containing 2–3 ml saline. It is then slowly inserted into one nostril until it touches the pharyngeal wall, and then the saline is quickly injected into the nostril, and the recoverable nasopharyngeal specimen is aspirated (43). The catheter is then withdrawn under suction. A labeled universal transport medium or sterile specimen container is used to store the aspirated fluid (44). Rodrigues* et al.* demonstrated that nasopharyngeal aspirates had higher sensitivity than naso‐oropharyngeal sampling in screening for COVID-19 (43). 

**Nasopharyngeal (NP) wash/aspiration samples in pediatric patients (babies):** Publications from China have introduced the nasopharyngeal swab as the COVID-19 diagnosis gold standard, even for children. The supine position may be used to collect the swab sample from the nasopharynx (45). 

It is safer for the caregiver to place themselves on the baby's side to decrease exposure to droplet projections. Children have a 6–7 cm nasal cavity, which is somewhat shorter than an adults. The swab may have a mark to show the correct insertion length. Before testing, each nostril may require the instillation of some drops of non-bacteriostatic saline (PH 7.0). The nasopharyngeal secretions may be aspirated by a piece of vacuum equipment fitted to a mucus trap through a probe or catheter (45). For each nostril, the catheter is probed parallel to the nasal floor to reach the nasopharynx. The catheter is rotated as it is pulled out slowly after the vacuum is activated. The sample may be limited to the probe or catheter, in which case a few drops of sterile saline may be required to aspirate the sample into the container (46). 

**Oropharyngeal swab: **The patient is asked to open their mouth wide open. The tongue is depressed using a wooden tongue depressor. Three separate contacts to the tonsillar areas and the pharynx are necessary for the swab to collect the necessary sample. The tongue should be avoided as it makes the sample less sensitive (39, 47). The oropharyngeal swab is less sensitive in detecting SARS-CoV-2 RNA than nasopharyngeal swabs (18). Studies have revealed that with a slightly lower SARS-CoV-2 RNA concentration, oropharyngeal swabs, and throat washings can be considered to detect SARS-CoV-2, and nasopharyngeal swabs show more diagnostic sensitivities and are less tolerated by patients (39, 48).

**Oral fluid collection: **This type of sampling varies widely from posterior oropharyngeal fluids or “deep throat” saliva with secretions from the oropharynx, saliva collected with special sponges or a pipet, or samples collected from drooling or spitting. Another method is collecting saline solutions after they have been gargled. A meta-analysis suggests that saliva is at best slightly less sensitive or similar to other specimens, including NP swabs (49, 50). Although oral fluid collection is a suitable and convenient method for the diagnosis of SARS-CoV-2 at the time of onset of symptoms, especially in outpatient populations, it is not an effective method (51).

**Mid-turbinate (MT) samples:** A nylon-flocked swab is inserted in the horizontal position until gentile resistance is met. Then the swab is left in for 10–15 seconds on each side, and then it is rotated. The process is repeated for the other nostril with the same swab (52). MT self-collection swabs are sensitive and are transported dry, making them an easy-to-use sampling method for the diagnosis of SARS-COV-2 (53). Jamal* et al.* demonstrated nasopharyngeal/MT swabs to have 15% greater sensitivity than MT swabs for SARS-CoV-2 detection in the first week of the disease, but they should be approached with caution (54). 

**Saliva**: The non-invasive collection of saliva reduces 2019-nCoV transmission risk making it ideal in cases of nasopharyngeal sampling contraindication or in cases where asymptomatic individuals need to be repeatedly screened (39). Wyllie AL* et al.* reported that salivary specimens are more sensitive than nasopharyngeal specimens for the detection of the COVID-19 virus (55). Other techniques for the collection of saliva that probably impact the method sensitivity include the early morning posterior oropharyngeal spitting technique where posterior oropharyngeal saliva is coughed up by throat clearing and spat into a sterile container, the general spitting technique, the drooling technique (unstimulated whole saliva), the posterior pharyngeal spitting technique, which is produced through coughing or clearing the throat and contains both lower and upper tract secretions, or a saliva collection device that collects saliva in the mouth floor. Sample quality variability can be reduced using a sample collection protocol that includes guidelines on abstention from eating and timing (56). In pediatric tertiary care hospitals, the saliva test has less sensitivity compared to nasopharyngeal and oropharyngeal swabs for SARS-CoV-2 diagnosis (57). The new SaliVISION assay, which is a rapid saliva-based SARS-CoV-2 screening, has higher sensitivity (98.28%) and specificity (100%) compared to either testing platform (58). 

**Lower Respiratory Tract Specimens (Sputum, Endotracheal Aspirate, or Bronchoalveolar Lavage):**

There is little evidence of lower respiratory tract sampling. The lower respiratory tract sample could be an option for patients who received invasive mechanical ventilation or those who have developed a productive cough (59). According to experiences in MERS-CoV, lower respiratory tract sample sensitivity may be higher than upper tract samples making it the preferred specimen type due to its higher viral loads (59). Collection of the lower respiratory samples (usually BALF) requires both a suction device and a skilled operator and is an invasive procedure. Therefore, BALF samples are not feasible for routine laboratory diagnosis and monitoring of COVID-19 (12).

Lower tract specimens, including tracheal aspirates, sputum, BAL specimens, intubation specimens, and productive cough specimens are collected from patients with severe symptoms. When possible, testing oropharyngeal and nasopharyngeal swabs is also advisable. Lower tract samples, such as sputum, BAL, and tracheal aspirate specimens, are indicated for patients in need of further testing, such as patients with negative oropharyngeal and nasopharyngeal swab tests and those with pneumonia (12, 42). 

**Sputum**

Sterile 50-mL plastic tubes are used to collect lower airway sputum. The quality of sputum samples is checked microscopically; if less than ten squamous epithelial cells and > 25 polymorphonuclear cells (PMNs) per low-power field are seen, the sample is regarded as high quality (60). 

Han* et al.* suggested that in patients who do not have a productive cough, sputum can be induced and collected by inhalation of 10 mL of 3% hypertonic saline through a mask with oxygen at a flow rate of 6 L/min for 20 min until sputum is produced (61). Recent studies on SARS-CoV-2 respiratory sample viral loads have shown sputum specimens to carry larger viral loads compared to throat swabs (36, 60, 62). Difficulty in producing sputum, for instance in elderly individuals, may limit its use for many with COVID-19 (60). 

It should be noted that extracting nucleic acids is very difficult while working with sputum samples with high viscosity (60). Therefore, reliable test results may require homogenizing and mixing the sputum samples (60, 63). Despite the current lack of standardized pre-treatment procedures for COVID-19 detection, Hong* et al.* found that for sputum homogenization, the use of N-acetyl-cysteine dissolved in a sodium citrate solution is both an effective and feasible procedure (1). In Lin* et al.*'s study, sputum samples completely liquefied by 30 min shaking at room temperature with a similar amount of N-acetyl cysteine (10 g/L) were used for the assay (60). A swab can be used to add 500 μL sputum to a 2-mL microtube, which should be then mixed with a similar volume of viral transport media or (VTM) PBS using glass beads, vortexed and centrifuged sufficiently, and the supernatant used for extraction to achieve adequate results (1). A study showed that rapid antigen testing and sputum testing have low sensitivity compared to sputum testing (60). A meta-analysis revealed that nasopharyngeal swabs, saliva, and deep throat sputum yielded the same results, with their sensitivity directly influenced by the severity of the disease (64).

**Blood, Plasma, Serum**

SARS-CoV-2 RNA detection in blood samples has been reported in some patients, and some studies suggest that detection in the blood is associated with disease severity (52, 65). Whole blood and serum can be stored in the refrigerator (2–8°C) for less than 5 days and should be kept in the freezer (–70°C on dry ice) for a longer time (66). Hogan* et al.*’s study demonstrated a high likelihood of RNA virus detection in the blood of ICU patients under ventilation (67). COVID-19 viremia has been reported in 26% (42/159) of the serum samples and 15% (11/71) of the blood samples in Peng* et al.*’s study (68). SARS-CoV-2 nucleocapsid protein (NP) in the blood is a rapid, convenient, and accurate method for detecting SARS-CoV-2 in hospitalized patients (69). 

**Urine**

The urine-based ELISA (enzyme-linked immunosorbent assay) is a noninvasive and facile COVID-19 immunodiagnostic platform that can screen anti-SARS-CoV-2 antibodies (70). However, SARS-CoV-2 RNA levels in urine are quite small and the studies conducted in this field are limited (71). The number of patients with positive urine samples for SARS-CoV-2 RNA was 1 out of 3 (33.3%) among severe, 1 out of 8 (12.5%) among moderate, and 0 out of 9 (0%) among mild cases. Compared to other samples such as stools (14–18 days) and pharyngeal swabs (up to 30 days), SARS-CoV2 RNA may be detected in the urine for a relatively short period of at least 4 days (72). Despite urinary viral shedding for a short period after infection, while working with urine samples, especially from patients with moderate to severe COVID-19, infection prevention measures and instructions should be observed (20). Urine samples, like blood and serum samples, can be stored for less than 5 days in the refrigerator (2–8°C) in a urine collection container and should be kept in the freezer (–70°C on dry ice) for a longer time (20). 

**Stool and Anal Swab**

Stool and anal swabs have high viral load and early positive detection, but these samples can have a greater risk of contamination (73). One study found that respiratory specimens have higher viral loads than samples collected from the rectum (74). In cases with negative URT and LRT, if there is still clinical suspicion of a COVID-19 infection, NAAT can be considered for fecal specimens in the second week of symptoms. When testing feces, it is necessary to validate the power of the intended extraction method and NAAT for this sample type (75). Although the infectivity of RNA-positive SARS-CoV-2 stool is unknown, Wu* et al.* suggested that viral shedding in the stool lasts approximately 2–4 weeks. They observed that in over half of the patients, fecal samples remained positive for SARS-CoV-2 RNA for a mean of 11.2 days after respiratory tract samples became negative (76). The virus may be detected in the stool after diarrhea has stopped (77). The stool should be collected in a sterile plain bottle and 0.1 g of it suspended in a 1 mL viral transport medium (1:10 dilution) (76) and can be stored in the refrigerator (2–8°C) for less than 5 days and should be kept in the freezer (–70°C on dry ice) for a longer time (20). Then the sample should be centrifuged at 4000 g for 20 min and a 140 µL aliquot of the filtrate prepared for later use (76). The comparison between the 2019-nCoV nucleic acid tests in stool samples and anal swabs showed a 9.83% positive rate in feces and a 10.00% positive rate in anal swabs (78).

**Semen**

No comprehensive data are available addressing the infectivity of semen and vaginal secretions. Li* et al.* showed the semen of men with COVID-19 and that of recovering patients to contain SARS-CoV-2, highlighting the possibility that monitoring transmission through sex might be an important step in disease prevention, especially because recovering patients showed they may be able to transmit the virus through their semen (79). Semen samples for SARS-CoV-2 detection were obtained by masturbation, and all of the ejaculates were collected into a sterile wide-mouthed calibrated container after an asexual abstinence period of two to five days (80). A study reported that SARS-CoV-2 was rarely present in semen during the acute phase of COVID-19. This type of contamination can be associated with mouth or hand contamination during semen collection (81). Saylam* et al.* in their study revealed that infected men who have high viral load and severe clinical conditions may be able to transmit SARS‐CoV‐2 in their semen samples (82).

**Ocular Fluid**

Ocular symptoms are present as the initial signs of infection. Therefore, we cannot completely exclude the eye as a potential source of infection (83). A study showed that SARS-CoV-2 was not founded by RT-PCR in ocular fluid (aqueous or vitreous humor) (84). Both patients with and without signs of conjunctivitis can have detectable SARS-CoV-2 in their ocular fluids (67). The conjunctival swabs were obtained from one of the affected eyes of patients with ocular symptoms or randomly from one eye of patients without ocular symptoms by two experienced physicians (73). The presence of SARS-CoV-2 in the conjunctival swabs implicates the ocular fluids as a potential source of the disease (85). 

**Other less Important Body Fluids**

Some body fluids do not have a high diagnostic accuracy based on laboratory tests. Due to severe inflammation, COVID‐19 patients’ conditions are occasionally complicated with extensive body cavity effusions, including pericardial effusion, pleural effusion, and peritoneal effusion (86). However, none of the available studies indicate the presence of the virus in pericardial effusion, peritoneal effusion, posterior fornix, joint fluid, peritoneal exudate, female reproductive tract secretions, or amniotic fluid (27). Evidence of a systematic review by Lewis,* et al.* showed that COVID-19 patients’ cerebrospinal fluid (CSF) does not have detectable SARS-CoV-2, even in those with neurological manifestations (87). 

**Post-mortem Specimens**

The CDC guidance for COVID-19 testing on postmortem specimens was updated on November 22, 2020, and it recommended that if an autopsy is performed on a suspected COVID-19 case, nasopharyngeal swab/NP swab, lung swab, and formalin-fixed lung autopsy tissues be collected. If an autopsy is performed within 2 hours of the death of a person not suspected of COVID-19, it is recommended that a postmortem nasopharyngeal swab (NP swab) be collected for COVID-19 testing (88, 89). 

Autopsies of confirmed COVID-19 patients should also include formalin-fixed autopsy tissues from upper airways and major organs, such as the lungs, and sample collection for testing of other respiratory pathogens and other postmortem infectious disease and microbiologic tests (89).

In the study by Basso* et al.*, using the best specific personal protective equipment (PPE), tissues and organs, such as the lungs, heart, liver, kidney, spleen, and brain, were harvested and fixed in 10% formalin solution for histopathologic examination (4% final formaldehyde concentration) as a whole or in fragments within rigid plastic containers. For molecular analysis and electron microscopic analysis, they used RNA later (RNA stabilization solution) and Karnovsky's fixative (paraformaldehyde-glutaraldehyde), respectively. Then, the RNA later and Karnovsky’s fixative stored in test tubes of different shapes or cap colors were used for specific tissue/organ samples. Lung tissue fragments preserved in saline phosphate buffer and endobronchial swabs were regularly sent for molecular and cultural analysis to the virology and microbiology laboratory (90).

**Swabs**

Several types of swabs have been used for sampling to detect coronavirus, including flocked nylon swabs, rayon swabs, spun polyester swabs, and cotton swabs (77). The WHO has not provided any reference procedures for collecting (neither lower nor higher) respiratory material for SARS-CoV-2 diagnosis. The CDC (Center for Disease Control and Prevention) recommends that synthetic-tipped swabs, such as Dacron or nylon swabs with plastic or aluminum shafts, be used to collect oropharyngeal and nasopharyngeal material and that lower respiratory tract samples be collected as soon as feasible or available (8). Wooden-shaft and calcium alginate swabs are not recommended as their substances may interfere with the amplification of nucleic acids (21, 77). Not all molecular platforms are guaranteed to be compatible with Rayon swabs. Swabs that have stoppers simplify distance estimation for MT self-collection, especially as pediatric patients have different swab insertion distances. MT sampling does not always need to be done on both sides (21).

After the specimen collection, the viral transport media tube should be labeled and the tight seal of the cap on the tube must be checked. 

**Timelines**

Although not well documented, sample collection timing based on the onset of symptoms is also essential (25). According to WHO guidelines, specimen collection should be done at the first chance after the onset of symptoms (within seven days if possible) and before antiviral medications are administered. Positive SARS-CoV-2 samples were found in Chinese patients 1 to 2 days before symptom onset and persisted for up to 2 weeks in severe cases (20). In a study, prolonged MERS-CoV RNA shedding in the respiratory tract was associated with diabetes (91). Viral load peaks at around one week to 10 days after the beginning of symptoms in patients with SARS-CoV infection, probably in connection with the nosocomial spread of the virus associated with healthcare workers (92)**. **

Although logistically difficult for healthcare workers and patients, symptom onset is the best time for taking swabs in COVID-19 as it is when viral load is at its highest. Swabs taken later than that may yield false negative results (18, 93), He* et al.* have estimated that viral shedding of patients with laboratory-confirmed COVID-19 peaks on or before symptom onset, and a substantial proportion of transmission probably occurs before the first symptoms (93). Yang* et al.* collected BALF, sputum, nasal swabs, and throat swabs 0–7, 8–14, and more than 15 days after disease onset. They found that in the first fortnight after disease onset, in addition to BALF, sputum samples presented the highest positivity rate (74.4% and 88.9%), followed by nasal swabs (53.6% and 73.3%) in severely and mildly symptomatic patients, respectively. Nasal and sputum swabs presented lower (61.1 % and 42.9%, respectively) positive rates fifteen days after disease onset (23). Kucirka LM,* et al.*'s study suggests the lowest rate of false negatives is recorded 3 days after the onset of symptoms or approximately 8 days after contact (94). 

**Transport Medium, Packing, Storage, and Transportation**

Two of the critical issues are the correct collection of samples and their correct transport to the laboratory (21). This stage is also related to the proper use of packaging materials, labeling, and reminders to reduce the likelihood of packages getting damaged, minimize exposure, and improve the carriers' efficiency and confidence in package delivery. When a delay in specimen delivery to the laboratory is likely, using a VTM (viral transport medium) is highly recommended (20).

Nasal and oropharyngeal swabs are recommended to be placed in a single sterile, screw-cap, leak-proof tube containing a VTM (2–3 ml) or sterile saline (60) between 2–8°C immediately after collection (8). If VTM is not available, dry swabs can be used, placed in a sterile tube, and sent at ambient temperature as long as they reach the laboratory within two days (8). However, in some studies, nasopharyngeal and oropharyngeal specimens are collected in sterile tubes either separately (8) or in combination in a viral transport medium (2–3 mL) placed in a single tube for each participant before RNA isolation (1, 8, 95).

Radbel J* et al.* claimed that phosphate-buffered saline (PBS) can be used for transport and short-term preservation of specimens containing SARS-CoV-2. It allows high intra-individual and inter-individual reliability and maintains viral stability compared with VTM in the detection of the three SARS-CoV-2 genes (N gene, ORF1ab, and S gene) through 18 hours of storage (96). Also, Rodino* et al.* demonstrated reliable detection of SARS-CoV-2 RNA in swabs stored in minimal essential medium (MEM), PBS, saline, and VTM after seven days at 2–8°C and frozen at -20°C (97). Endotracheal aspirate, bronchoalveolar lavage, or sputum in sterile sample tubes do not need a universal/viral transport medium (UTM). Guidelines for laboratory diagnosis of COVID-19 in Korea recommend immediate refrigeration at 2–4°C and freezing at -70°C of collected upper respiratory samples for less than and longer than five days, respectively until testing is carried out (1). 

In case it is necessary to transport the specimens to a referral laboratory, they must be kept cool and flocked or Dacron swabs placed in VTM should be maintained at 2–8°C (98) in a cold box with ice packs. Quality control is required at all stages of sampling.

The World Health Organization (WHO) guidelines recommend a ‘triple packaging system’ to contain infectious material. This packaging consists of 1) a tightly sealed vacutainer (adsorbent material), 2) leak-proof packaging such as a falcon tube or a universal bottle where falcon tubes are not available, and 3) an external packaging layer protecting the second layer from physical damage in transport, especially for further processing of the specimens in another city or country (36). The outer packaging material (the third layer) should be very durable and be labeled ''Specimen for COVID-19 testing" (36).

The specimen should be tightly capped and locked in a biohazard container or zip-lock bag placed inside a leak-proof cryobox with an easily observable biohazard label and transported at room temperature or under cold conditions for short-distance or long-distance transport, respectively (10, 36).

**Potential Interfering Factors**

It is essential that the transport and packaging are appropriate for keeping the virus detectable and viable and that the sample quality is good, containing adequate secretions and cells (99).

Extracted viral RNA has been reported to lose stability as a result of pre-analytical inconsistencies (9, 100). An important cause of false-negative COVID-19 results alongside low viral load is RNA instability. Processing problems and transport delays between sampling and extraction may lead to increased false negativity. Swab tubes containing RNA stabilization medium enable the laboratory to batch-test the samples without compromising RNA integrity; a multicenter study has validated adding RNA stabilization reagent and has demonstrated a reduction in RT-PCR assay failure rates and improvement in integrity and yield for external quality assessment schemes (100). A 41% false-negative rate has been reported for RT-PCR in COVID-19, and there are reports of positive results on test repeats for initially swab-negative patients (9). Because sore throat is one of the main symptoms of influenza but not a symptom of COVID-19, the virion levels in COVID-19 naso/oropharyngeal (NOP) specimens may be much lower than they are in influenza (9). Thus, SARS-CoV-2 infection is not completely ruled out by a negative result does not rule out the possibility of a SARS-CoV-2 infection; several factors, including poor quality of samples, the inadequacy of sampling (67, 99), inappropriate handling during shipment or storage, too early or late collection of samples, and inherent technical test flaws, such as PCR inhibition or virus mutation, could cause false-negative results (99). Because an uninfected population is yet to be tested, our data is not accurate enough to measure false-positive rates. However, the chances of false-positive results are inherently low in the PCR design (9). 

**Safe Specimen Collection and Handling**

One of the challenges in sampling is the contamination of health workers and the direct handling of patient samples. In general, these problems can be prevented by following the safety standards and guidelines, training the personnel, and the methods patients use to take samples from themselves. Researchers believe that the coronavirus can survive in the air and on surfaces and cause infection for a long time (101). Safety measures should be taken in sample collection, transport and shipping, sample processing, and studies on the virus (4). Hygienic collection of samples should be done without the involvement of third parties to prevent any potential risk of infection, provided that the sample can be safely processed through a safe collection system (102). Any stage of the process, including sample collection, transport, processing, and disposal can infect the workers with COVID-19 (36, 103). WHO guidelines dictate that every specimen (feces, blood, body fluids, and swabs) can be infectious (20). In general, health and safety measures should include biosafety training and awareness programs for laboratory workers, and biosafety measures during specimen collection, transport, and processing (1, 36, 103). 

A biological safety cabinet should be used in all initial processing stages and specimen handling before inactivation. A Biological Safety Level-2 (BSL-2) laboratory (103, 104) is necessary to conduct molecular testing. Suitably trained and competent personnel should be in charge of viral culture and isolation, and the procedure must take place in a BSL-3 laboratory (16). The necessary PPE (personal protective equipment (PPE) requirements for sampling personnel include double-layer latex gloves, protective clothing, waterproof boot covers, eye protection (visors or goggles), and N95 masks or masks with higher filtration efficiency; regular changing of the latex glove outer layer is necessary, should sampling personnel touch patient secretions, body fluids, blood, etc. (1, 27). As a general laboratory protocol, laboratory waste and all used personal protective equipment (PPE) generated should be referred to a biomedical waste management facility and decontaminated before disposal (36).

## Conclusion

Although, in this review, SARS-CoV-2 was detected in non-airborne body fluids, the lower and upper respiratory tract specimens still are associated with a higher accuracy form diagnostic standpoint, speed, and feasibility of testing in most societies. However, choice of the type of diagnostic test can vary based on the patient's condition, the duration of the disease, and the purpose of the diagnostic test. 

Explanation of the diagnostic accuracy of different sample types is crucial for laboratory diagnosis and monitoring of SARS-CoV-2. Diagnosis of viral infection is greatly dependent on proper sampling. Inadequate specimen collection, either it is from confirmed or suspected COVID-19 patients, may lead to inconclusive results and misdiagnosis. Thus, all clinical laboratories require standard operating procedures (SOPs) for sampling. In addition, healthcare providers should consider continuing protection and following safety guidelines, even after patients show relief in symptoms. Given the consideration of a wide spectrum of the objectives of the study, a large number of studies were included in this study. One of the limitations of this study was lack of observational studies on a larger scale, which needs to be considered in future research.

## Funding

None.

## Conflict of Interest

None.

## References

[1] Hong KH, Lee SW, Kim TS, Huh HJ, Lee J, Kim SY (2020). Guidelines for Laboratory Diagnosis of Coronavirus Disease 2019 (COVID-19) in Korea. Ann Lab Med..

[2] Li Q, Guan X, Wu P, Wang X, Zhou L, Tong Y (2020). Early Transmission Dynamics in Wuhan, China, of Novel Coronavirus-Infected Pneumonia. N Engl J Med..

[3] Jerving S (2021). The long road ahead for COVID-19 vaccination in Africa. Lancet (London, England)..

[4] Nyaruaba R, Mwaliko C, Hong W, Amoth P, Wei H (2021). SARS-CoV-2/COVID-19 Laboratory Biosafety Practices and Current Molecular Diagnostic Tools. Int. J Biosaf Biosecurity.

[5] Guo YR, Cao QD, Hong ZS, Tan YY, Chen SD, Jin HJ (2020). The origin, transmission and clinical therapies on coronavirus disease 2019 (COVID-19) outbreak - an update on the status. Mil Med Res..

[6] La Marca A, Capuzzo M, Paglia T, Roli L, Trenti T, Nelson SM (2020). Testing for SARS-CoV-2 (COVID-19): a systematic review and clinical guide to molecular and serological in-vitro diagnostic assays. Reprod Biomed Online..

[7] Ahn DG, Shin HJ, Kim MH, Lee S, Kim HS, Myoung J (2020). Current Status of Epidemiology, Diagnosis, Therapeutics, and Vaccines for Novel Coronavirus Disease 2019 (COVID-19). J Microbiol Biotechnol..

[8] Lippi G, Simundic AM, Plebani M (2020). Potential preanalytical and analytical vulnerabilities in the laboratory diagnosis of coronavirus disease 2019 (COVID-19). Clin Chem Lab Med..

[9] Payne D, Newton D, Evans P, Osman H, Baretto R (2021). Preanalytical issues affecting the diagnosis of COVID-19. J Clin Pathol..

[10] Tan SS, Yan B, Saw S, Lee CK, Chong AT, Jureen R (2021). Practical laboratory considerations amidst the COVID-19 outbreak: early experience from Singapore. J Clin Pathol..

[11] Kojima N, Turner F, Slepnev V, Bacelar A, Deming L, Kodeboyina S, Klausner JD (2021). Self-collected oral fluid and nasal swab specimens demonstrate comparable sensitivity to clinician-collected nasopharyngeal swab specimens for the detection of SARS-CoV-2. Clin Infect..

[12] Chadaga K, Chakraborty C, Prabhu S, Umakanth S, Bhat V, Sampathila N (2022). Clinical and Laboratory Approach to Diagnose COVID-19 Using Machine Learning. Interdiscip. Sci..

[13] Hong KH, Kim GJ, Roh KH, Sung H, Lee J, Kim SY (2022). Update of Guidelines for Laboratory Diagnosis of COVID-19 in Korea. Ann Lab Med..

[14] Kukull B, Shakir SM, Hanson KE (2022). Performance of Non-nasopharyngeal Sample Types for Molecular Detection of SARS-CoV-2. Clin Lab Med..

[15] Caruana G, Croxatto A, Coste AT, Opota O, Lamoth F, Jaton K (2020). Diagnostic strategies for SARS-CoV-2 infection and interpretation of microbiological results. Clin Microbiol Infect..

[16] Stanoeva KR, van der Eijk AA, Meijer A, Kortbeek LM, Koopmans MPG, Reusken C (2021). Towards a sensitive and accurate interpretation of molecular testing for SARS-CoV-2: a rapid review of 264 studies. Euro Commun Dis Bulletin..

[17] Zhang Y, Cen M, Hu M, Du L, Hu W, Kim JJ (2021). Prevalence and Persistent Shedding of Fecal SARS-CoV-2 RNA in Patients With COVID-19 Infection: A Systematic Review and Meta-analysis. Clinical Transl Gastroenterol..

[18] Wölfel R, Corman VM, Guggemos W, Seilmaier M, Zange S, Müller MA (2020). Virological assessment of hospitalized patients with COVID-2019. Nature..

[19] Rong G, Zheng Y, Chen Y, Zhang Y, Zhu P, Sawan M (2021). COVID-19 diagnostic methods and detection techniques: a review. Ref Modul Biomed Sci..

[20] World Health Organization (2020). Laboratory testing for coronavirus disease (‎COVID-19)‎ in suspected human cases: interim guidance, 19 March 2020.

[21] Hanson KE, Altayar O, Caliendo AM, Arias CA, Englund JA, Hayden MK (2021). The Infectious Diseases Society of America Guidelines on the Diagnosis of COVID-19: Antigen Testing. Clinical Infect Dis.

[22] Sutjipto S, Lee PH, Tay JY, Mendis SM, Abdad MY, Marimuthu K (2020). The Effect of Sample Site, Illness Duration, and the Presence of Pneumonia on the Detection of SARS-CoV-2 by Real-time Reverse Transcription PCR. Open Forum Infect Dis..

[23] Yang Y, Yang M, Shen C, Wang F, Yuan J, Li J (2020). Evaluating the accuracy of different respiratory specimens in the laboratory diagnosis and monitoring the viral shedding of 2019-nCoV infections. MedRxiv.

[24] Chen X, Xia S (2022). Sensitive methods for detection of SARS-CoV-2 RNA. Methods Microbiol..

[25] Péré H, Podglajen I, Wack M, Flamarion E, Mirault T, Goudot G (2020). Nasal Swab Sampling for SARS-CoV-2: a Convenient Alternative in Times of Nasopharyngeal Swab Shortage. J Clin Microbiol..

[26] Lim J, Lee J (2020). Current laboratory diagnosis of coronavirus disease 2019. Korean J Intern Med..

[27] Loeffelholz MJ, Tang YW (2020). Laboratory diagnosis of emerging human coronavirus infections - the state of the art. Emerging microbes & infections..

[28] Miranda-Ortiz H, Fernández-Figueroa EA, Ruíz-García EB, Muñoz-Rivas A, Méndez-Pérez A, Méndez-Galván J (2021). Development of an alternative saliva test for diagnosis of SARS-CoV-2 using TRIzol: Adapting to countries with lower incomes looking for a large-scale detection program. PloS One..

[29] Premraj A, Aleyas AG, Nautiyal B, Rasool TJ (2020). Nucleic Acid and Immunological Diagnostics for SARS-CoV-2: Processes, Platforms and Pitfalls. Diagnostics (Basel, Switzerland)..

[30] Banada P, Elson D, Daivaa N, Park C, Desind S, Montalvan I (2021). Sample collection and transport strategies to enhance yield, accessibility, and biosafety of COVID-19 RT-PCR testing. J Med Microbiol..

[31] Cockerill FR, Wohlgemuth JG, Radcliff J, Sabol CE, Kapoor H, Dlott JS (2021). Evolution of Specimen Self-Collection in the COVID-19 Era: Implications for Population Health Management of Infectious Disease. Population Health Manag..

[32] Rasool G, Riaz M, Abbas M, Fatima H, Qamar MM, Zafar F (2022). COVID-19: Clinical laboratory diagnosis and monitoring of novel coronavirus infected patients using molecular, serological and biochemical markers: A review. Int J Immunopathol Pharmacol..

[33] Kretzschmar ME, Rozhnova G, Bootsma MCJ, van Boven M, van de Wijgert J, Bonten MJM (2020). Impact of delays on effectiveness of contact tracing strategies for COVID-19: a modelling study. Lancet Public Health..

[34] Grassly NC, Pons-Salort M, Parker EPK, White PJ, Ferguson NM (2020). Comparison of molecular testing strategies for COVID-19 control: a mathematical modelling study. Lancet Infect Dis..

[35] Humphreys DP, Gavin KM, Olds KM, Bonaca MP, Bauer TA (2022). At-home sample collection is an effective strategy for diagnosis and management of symptomatic and asymptomatic SARS-CoV-2 carriers. BMC Infect Dis..

[36] Karthik K, Aravindh Babu RP, Dhama K, Chitra MA, Kalaiselvi G, Alagesan Senthilkumar TM (2020). Biosafety Concerns During the Collection, Transportation, and Processing of COVID-19 Samples for Diagnosis. Arch Med Res..

[37] Callahan C, Lee R, Lee G, Zulauf KE, Kirby JE, Arnaout R (2020). Nasal-Swab Testing Misses Patients with Low SARS-CoV-2 Viral Loads. medRxiv.

[38] Hiebert NM, Chen BA, Sowerby LJ (2021). Variability in instructions for performance of nasopharyngeal swabs across Canada in the era of COVID-19 - what type of swab is actually being performed?. J otolaryngology - Head Neck Surg..

[39] Lee RA, Herigon JC, Benedetti A, Pollock NR, Denkinger CM (2021). Performance of Saliva, Oropharyngeal Swabs, and Nasal Swabs for SARS-CoV-2 Molecular Detection: a Systematic Review and Meta-analysis. J Clin Microbiol..

[40] Lee S, Widyasari K, Yang HR, Jang J, Kang T, Kim S (2022). Evaluation of the Diagnostic Accuracy of Nasal Cavity and Nasopharyngeal Swab Specimens for SARS-CoV-2 Detection via Rapid Antigen Test According to Specimen Collection Timing and Viral Load. Diagnostics (Basel, Switzerland)..

[41] Patriquin G, LeBlanc JJ, Williams C, Hatchette TF, Ross J, Barrett L (2022). Comparison between Nasal and Nasopharyngeal Swabs for SARS-CoV-2 Rapid Antigen Detection in an Asymptomatic Population, and Direct Confirmation by RT-PCR from the Residual Buffer. Microbiol Spectr..

[42] Kim DH, Kim D, Moon JW, Chae SW, Rhyu IJ (2022). Complications of Nasopharyngeal Swabs and Safe Procedures for COVID-19 Testing Based on Anatomical Knowledge. J Korean Med Sci..

[43] Rodrigues J, Gouveia C, Santos MA, Costa O, Côrte-Real R, Brito MJ (2021). Comparison of nasopharyngeal samples for SARS-CoV-2 detection in a paediatric cohort. J Pediatr Child Health..

[44] Garnett L, Bello A, Tran KN, Audet J, Leung A, Schiffman Z (2020). Comparison analysis of different swabs and transport mediums suitable for SARS-CoV-2 testing following shortages. J Virol Methods..

[45] Pondaven-Letourmy S, Alvin F, Boumghit Y, Simon F (2020). How to perform a nasopharyngeal swab in adults and children in the COVID-19 era. Euro Ann Otorhinolaryngol Head Neck Dis..

[46] Kaufman AC, Brewster R, Rajasekaran K (2020). How to Perform a Nasopharyngeal Swab - An Otolaryngology Perspective. Am J Med..

[47] Petruzzi G, De Virgilio A, Pichi B, Mazzola F, Zocchi J, Mercante G (2020). COVID-19: Nasal and oropharyngeal swab. Head Neck..

[48] Hitzenbichler F, Bauernfeind S, Salzberger B, Schmidt B, Wenzel JJ (2021). Comparison of Throat Washings, Nasopharyngeal Swabs and Oropharyngeal Swabs for Detection of SARS-CoV-2. Viruses..

[49] Teo AKJ, Choudhury Y, Tan IB, Cher CY, Chew SH, Wan ZY (2021). Saliva is more sensitive than nasopharyngeal or nasal swabs for diagnosis of asymptomatic and mild COVID-19 infection. Sci Rep..

[50] Williams E, Bond K, Zhang B, Putland M, Williamson DA (2020). Saliva as a Noninvasive Specimen for Detection of SARS-CoV-2. J Clin Microbiol..

[51] Manabe YC, Reuland C, Yu T, Azamfirei R, Hardick JP, Church T (2021). Self-Collected Oral Fluid Saliva Is Insensitive Compared With Nasal-Oropharyngeal Swabs in the Detection of Severe Acute Respiratory Syndrome Coronavirus 2 in Outpatients. Open Forum Infect Dis..

[52] Tu Y, Jennings R, Hart B, Cangelosi G, Wood R, Wehber K (2020). Patient-collected tongue, nasal, and mid-turbinate swabs for SARS-CoV-2 yield equivalent sensitivity to health care worker collected nasopharyngeal swabs. MedRxiv.

[53] D'Andrea EL, Cossu AM, Scrima M, Messina V, Iuliano P, Di Perna F (2021). Efficacy of Unsupervised Self-Collected Mid-Turbinate FLOQSwabs for the Diagnosis of Coronavirus Disease 2019 (COVID-19). Viruses..

[54] Jamal AJ, Mozafarihashjin M, Coomes E, Anceva-Sami S, Barati S, Crowl G (2021). Sensitivity of midturbinate versus nasopharyngeal swabs for the detection of severe acute respiratory syndrome coronavirus 2 (SARS-CoV-2). Infect Control Hospital Epidemiol..

[55] Wyllie AL, Fournier J, Casanovas-Massana A, Campbell M, Tokuyama M, Vijayakumar P (2020). Saliva is more sensitive for SARS-CoV-2 detection in COVID-19 patients than nasopharyngeal swabs. MedRxiv.

[56] Azzi L, Maurino V, Baj A, Dani M, d'Aiuto A, Fasano M (2021). Diagnostic Salivary Tests for SARS-CoV-2. J Dental Res..

[57] Ana Laura GO, Abraham Josué NR, Briceida LM, Israel PO, Tania AF, Nancy MR (2021). Sensitivity of the Molecular Test in Saliva for Detection of COVID-19 in Pediatric Patients With Concurrent Conditions. Front Pediatr..

[58] DeFina SM, Wang J, Yang L, Zhou H, Adams J, Cushing W (2022). SaliVISION: a rapid saliva-based COVID-19 screening and diagnostic test with high sensitivity and specificity. Sci Rep..

[59] Thwe PM, Ren P (2021). Analysis of sputum/tracheal aspirate and nasopharyngeal samples for SARS-CoV-2 detection by laboratory-developed test and Panther Fusion system. Diagn Microbiol Infect Dis..

[60] Lin C, Xiang J, Yan M, Li H, Huang S, Shen C (2020). Comparison of throat swabs and sputum specimens for viral nucleic acid detection in 52 cases of novel coronavirus (SARS-Cov-2)-infected pneumonia (COVID-19). Clin Chem Lab Med..

[61] Han H, Luo Q, Mo F, Long L, Zheng W (2020). SARS-CoV-2 RNA more readily detected in induced sputum than in throat swabs of convalescent COVID-19 patients. The Lancet Infect Dis..

[62] Saleem SM, Bhattacharya S (2022). Sputum Testing as the New Mass Screening Method for COVID-19 Patients in India - A Public Health Perspective. Int J Prevent Med..

[63] Fujimoto D, Fukuya M, Terao S, Irei I, Akiyama T, Watanabe A (2022). Sputum characteristics of patients with severe COVID-19: report of two cases with immunocytochemical detection of SARS-CoV-2 spike protein.

[64] Khiabani K, Amirzade-Iranaq MH (2021). Are saliva and deep throat sputum as reliable as common respiratory specimens for SARS-CoV-2 detection? A systematic review and meta-analysis. Am J Infect Control..

[65] Chiem C, Alghamdi K, Nguyen T, Han JH, Huo H, Jackson D (2022). The impact of COVID-19 on blood transfusion services: a systematic review and meta-analysis. Transfus Med Hemother..

[66] Xiao D, John Ling KH, Tarnowski T, Humeniuk R, German P, Mathias A (2021). Validation of LC-MS/MS methods for determination of remdesivir and its metabolites GS-441524 and GS-704277 in acidified human plasma and their application in COVID-19 related clinical studies. Anal Biochem..

[67] Hogan CA, Stevens BA, Sahoo MK, Huang C, Garamani N, Gombar S (2021). High Frequency of SARS-CoV-2 RNAemia and Association With Severe Disease. Clin Infect Dis..

[68] Peng L, Liu J, Xu W, Luo Q, Chen D, Lei Z (2020). SARS-CoV-2 can be detected in urine, blood, anal swabs, and oropharyngeal swabs specimens. J Med Virol..

[69] Thudium RF, Stoico MP, Høgdall E, Høgh J, Krarup HB, Larsen MAH (2021). Early Laboratory Diagnosis of COVID-19 by Antigen Detection in Blood Samples of the SARS-CoV-2 Nucleocapsid Protein. J Clin Microbiol..

[70] Ludolf F, Ramos FF, Bagno FF, Oliveira-da-Silva JA, Reis TAR, Christodoulides M (2022). Detecting anti-SARS-CoV-2 antibodies in urine samples: A noninvasive and sensitive way to assay COVID-19 immune conversion. Sci Adv..

[71] García-Bernalt Diego J, Fernández-Soto P, Muñoz-Bellido JL, Febrer-Sendra B, Crego-Vicente B, Carbonell C (2021). Detection of SARS-CoV-2 RNA in Urine by RT-LAMP: A Very Rare Finding. J Clin Med..

[72] Nomoto H, Ishikane M, Katagiri D, Kinoshita N, Nagashima M, Sadamasu K (2020). Cautious handling of urine from moderate to severe COVID-19 patients. Am J Infect Control..

[73] Wang Y, Chen X, Wang F, Geng J, Liu B, Han F (2021). Value of anal swabs for SARS-COV-2 detection: a literature review. Int J Med Sci..

[74] Jung SH, Kim SW, Lee H, Oh JH, Lim J (2021). Serial Screening for SARS-CoV-2 in Rectal Swabs of Symptomatic COVID-19 Patients. J Korean Med Sci..

[75] Sun M, Guo D, Zhang J, Zhang J, Teng HF, Xia J (2020). Anal swab as a potentially optimal specimen for SARS-CoV-2 detection to evaluate hospital discharge of COVID-19 patients. Future Microbiol..

[76] Ng SC, Chan FKL, Chan PKS (2020). Screening FMT donors during the COVID-19 pandemic: a protocol for stool SARS-CoV-2 viral quantification. Lancet Gastroenterol Hepatol..

[77] Tu YP, O'Leary TJ (2020). Testing for Severe Acute Respiratory Syndrome-Coronavirus 2: Challenges in Getting Good Specimens, Choosing the Right Test, and Interpreting the Results. Crit Care Med..

[78] Wu J, Liu J, Li S, Peng Z, Xiao Z, Wang X (2020). Detection and analysis of nucleic acid in various biological samples of COVID-19 patients. Travel Med Infect Dis..

[79] Li D, Jin M, Bao P, Zhao W, Zhang S (2020). Clinical Characteristics and Results of Semen Tests Among Men With Coronavirus Disease 2019. JAMA Netw Open..

[80] Temiz MZ, Dincer MM, Hacibey I, Yazar RO, Celik C, Kucuk SH (2021). Investigation of SARS-CoV-2 in semen samples and the effects of COVID-19 on male sexual health by using semen analysis and serum male hormone profile: A cross-sectional, pilot study. Andrologia..

[81] Delaroche L, Bertine M, Oger P, Descamps D, Damond F, Genauzeau E (2021). Evaluation of SARS-CoV-2 in semen, seminal plasma, and spermatozoa pellet of COVID-19 patients in the acute stage of infection. PloS One..

[82] Saylam B, Uguz M, Yarpuzlu M, Efesoy O, Akbay E, Çayan S (2021). The presence of SARS-CoV-2 virus in semen samples of patients with COVID-19 pneumonia. Andrologia..

[83] Petronio Petronio G, Di Marco R, Costagliola C (2020). Do Ocular Fluids Represent a Transmission Route of SARS-CoV-2 Infection?. Front Med..

[84] Hada M, Khilnani K, Vyas N, Chouhan JK, Dharawat KS, Bhandari S (2021). Evaluating the presence of SARS-CoV-2 in the intraocular fluid of COVID-19 patients. Indian J Ophthalmol..

[85] Zhong Y, Wang K, Zhu Y, Lyu D, Yu Y, Li S (2021). Ocular manifestations in COVID-19 patients: A systematic review and meta-analysis. Travel Med Infect Dis..

[86] Xia R, Hsu Lin L, Sun W, Moreira AL, Simsir A, Brandler TC (2022). Effusion fluid cytology and COVID-19 infection. Cancer Cytopathol..

[87] Lewis A, Frontera J, Placantonakis DG, Lighter J, Galetta S, Balcer L (2021). Cerebrospinal fluid in COVID-19: A systematic review of the literature. J Neurol Sci..

[88] d'Aleo F, Arghittu M, Bandi C, Conte M, Creti R, Farina C (2020). Guidance to post-mortem collection and storage of biological specimens for the diagnosis of Covid-19 infection. Microbiol Med..

[89] Centres for Disease Control and Prevention Collection and Submission of Postmortem Specimens from Deceased Persons with Known or Suspected COVID-19, March 2020(Interim Guidance).

[90] Basso C, Calabrese F, Sbaraglia M, Del Vecchio C, Carretta G, Saieva A (2020). Feasibility of postmortem examination in the era of COVID-19 pandemic: the experience of a Northeast Italy University Hospital. Int J Pathol..

[91] Al-Abdely HM, Midgley CM, Alkhamis AM, Abedi GR, Lu X, Binder AM (2019). Middle East Respiratory Syndrome Coronavirus Infection Dynamics and Antibody Responses among Clinically Diverse Patients, Saudi Arabia. Emerg Infect Dis..

[92] Mølhave M, Agergaard J, Wejse C (2022). Clinical Management of COVID-19 Patients - An Update. Seminars Nuclear Med..

[93] He X, Lau EHY, Wu P, Deng X, Wang J, Hao X (2020). Temporal dynamics in viral shedding and transmissibility of COVID-19. Nature Med..

[94] Kucirka LM, Lauer SA, Laeyendecker O, Boon D, Lessler J (2020). Variation in False-Negative Rate of Reverse Transcriptase Polymerase Chain Reaction-Based SARS-CoV-2 Tests by Time Since Exposure. Ann Intern Med.

[95] Gudbjartsson DF, Helgason A, Jonsson H, Magnusson OT, Melsted P, Norddahl GL (2020). Spread of SARS-CoV-2 in the Icelandic Population. N Engl J Med..

[96] Radbel J, Jagpal S, Roy J, Brooks A, Tischfield J, Sheldon M (2020). Detection of Severe Acute Respiratory Syndrome Coronavirus 2 (SARS-CoV-2) Is Comparable in Clinical Samples Preserved in Saline or Viral Transport Medium. J. Mol. Diagn..

[97] Rodino KG, Espy MJ, Buckwalter SP, Walchak RC, Germer JJ, Fernholz E (2020). Evaluation of Saline, Phosphate-Buffered Saline, and Minimum Essential Medium as Potential Alternatives to Viral Transport Media for SARS-CoV-2 Testing. J Clin Microbiol..

[98] Tang YW, Schmitz JE, Persing DH, Stratton CW (2020). Laboratory Diagnosis of COVID-19: Current Issues and Challenges. J Clin Microbiol..

[99] Coronavirus disease 2019 (COVID-19) caused by a Novel Coronavirus (SARS-CoV-2) Guidelines for case-finding, diagnosis, and public health response in South Africa.

[100] Malentacchi F, Pazzagli M, Simi L, Orlando C, Wyrich R, Günther K (2014). SPIDIA-RNA: second external quality assessment for the pre-analytical phase of blood samples used for RNA based analyses. PloS One..

[101] Habibi N, Behbehani M, Uddin S, Al-Salameen F, Shajan A, Zakir F A safe and effective sample collection method for assessment of SARS-CoV-2 in aerosol samples. InEnvironmental resilience and transformation in times of COVID-19.

[102] Majam M, Msolomba V, Scott L, Stevens W, Marange F, Kahamba T (2021). Self-Sampling for SARS-CoV-2 Diagnostic Testing by Using Nasal and Saliva Specimens: Protocol for Usability and Clinical Evaluation. JMIR Res Protoc..

[103] Naeem W, Zeb H, Rashid MI (2022). Laboratory biosafety measures of SARS-CoV-2 at containment level 2 with particular reference to its more infective variants. Biosaf Health..

[104] Bain W, Lee JS, Watson AM, Stitt-Fischer MS (2020). Practical Guidelines for Collection, Manipulation and Inactivation of SARS-CoV-2 and COVID-19 Clinical Specimens. Curr Protoc Cytom..

